# 4379 MARCKS protein is altered in naturally occurring model of asthma in horses

**DOI:** 10.1017/cts.2020.412

**Published:** 2020-07-29

**Authors:** Kaori Davis, Mary Sheats

**Affiliations:** 1 North Carolina State University

## Abstract

OBJECTIVES/GOALS: Asthma is a significant health concern that affects people of all ages worldwide. EAS demonstrates many of the pathophysiological characteristics of nonatopic human asthma, which has led EAS to be used as naturally occurring model. Previous work from our lab determined that MARCKS (Myristoylated Alanine Rich C Kinase Substrate) protein is an essential regulator of cellular inflammatory functions. In the current study, we hypothesized that MARCKS levels would be increased in BAL cell lysates from horses with EAS, and that inhibition of MARCKS in zymosan-stimulated BAL cells (*ex vivo*) would diminish respiratory burst. METHODS/STUDY POPULATION: Lysates were prepared from BAL cells isolated from horses with no, mild/moderate and severe EAS. Relative MARCKS protein levels were determined using equine specific MARCKS ELISA (MyBioSource). Cultured BAL cells were pretreated with a MARCKS inhibitor peptide (MANS), control peptide (RNS) or vehicle control and stimulated with zymosan for 5 hours. Reactive oxygen species levels were determined by luminescence to evaluate respiratory burst. Data were analyzed by One-way ANOVA (*p*<0.05). RESULTS/ANTICIPATED RESULTS: We determined that normalized MARCKS protein expression is significantly increased in BAL cell lysates from horses with mild/moderate or severe EAS, compared to horses with normal BAL cytology. Preliminary findings also suggest that MANS treatment of zymosan-stimulated equine BAL cells *ex vivo* attenuates levels respiratory burst. DISCUSSION/SIGNIFICANCE OF IMPACT: These findings point to a possible role for MARCKS protein in the pathophysiology of EAS and support MARCKS inhibition as a potential therapeutic strategy.

